# *Panax*-derived Zingibroside R1 exerts allelopathic effects on root architecture by modulating the auxin transport pathway and oxidative stress responses

**DOI:** 10.1093/hr/uhag185

**Published:** 2026-05-08

**Authors:** Xinjie Yang, Yihan Ye, Weipeng Lian, Xiang Li, Jian Yang, Yonghong Zhang, Lei You, Lanlan Zheng, Yun Zhu, Jiří Friml, Eva Benková, Chen Li, Luqi Huang, Juan Liu

**Affiliations:** School of Pharmacy, Shaanxi University of Chinese Medicine, Xianyang, Shaanxi 712046, China; School of Pharmacy, Shaanxi University of Chinese Medicine, Xianyang, Shaanxi 712046, China; Shiyan Key Laboratory of Medicinal Plants and Evolutionary Genetics, Hubei Key Laboratory of Embryonic Stem Cell Research, School of Basic Medicine, Hubei University of Medicine, Shiyan 442000, China; State Key Laboratory for Quality Ensurance and Sustainable Use of Dao-di Herbs, National Resource Center for Chinese Materia Medica, China Academy of Chinese Medical Sciences, Beijing 100700, China; Shiyan Key Laboratory of Medicinal Plants and Evolutionary Genetics, Hubei Key Laboratory of Embryonic Stem Cell Research, School of Basic Medicine, Hubei University of Medicine, Shiyan 442000, China; State Key Laboratory for Quality Ensurance and Sustainable Use of Dao-di Herbs, National Resource Center for Chinese Materia Medica, China Academy of Chinese Medical Sciences, Beijing 100700, China; School of Pharmacy, Shihezi University, Key Laboratory of Xinjiang Phytomedicine Resource and Utilization, Ministry of Education, Xinjiang, Shihezi 832000, China; State Key Laboratory for Quality Ensurance and Sustainable Use of Dao-di Herbs, National Resource Center for Chinese Materia Medica, China Academy of Chinese Medical Sciences, Beijing 100700, China; State Key Laboratory for Quality Ensurance and Sustainable Use of Dao-di Herbs, National Resource Center for Chinese Materia Medica, China Academy of Chinese Medical Sciences, Beijing 100700, China; Shiyan Key Laboratory of Medicinal Plants and Evolutionary Genetics, Hubei Key Laboratory of Embryonic Stem Cell Research, School of Basic Medicine, Hubei University of Medicine, Shiyan 442000, China; Shiyan Key Laboratory of Medicinal Plants and Evolutionary Genetics, Hubei Key Laboratory of Embryonic Stem Cell Research, School of Basic Medicine, Hubei University of Medicine, Shiyan 442000, China; Shiyan Key Laboratory of Medicinal Plants and Evolutionary Genetics, Hubei Key Laboratory of Embryonic Stem Cell Research, School of Basic Medicine, Hubei University of Medicine, Shiyan 442000, China; School of Pharmacy, Shihezi University, Key Laboratory of Xinjiang Phytomedicine Resource and Utilization, Ministry of Education, Xinjiang, Shihezi 832000, China; Institute of Science and Technology Austria, Am Campus 1, Klosterneuburg 3400, Austria; Institute of Science and Technology Austria, Am Campus 1, Klosterneuburg 3400, Austria; Shiyan Key Laboratory of Medicinal Plants and Evolutionary Genetics, Hubei Key Laboratory of Embryonic Stem Cell Research, School of Basic Medicine, Hubei University of Medicine, Shiyan 442000, China; State Key Laboratory for Quality Ensurance and Sustainable Use of Dao-di Herbs, National Resource Center for Chinese Materia Medica, China Academy of Chinese Medical Sciences, Beijing 100700, China; School of Pharmacy, Shaanxi University of Chinese Medicine, Xianyang, Shaanxi 712046, China; State Key Laboratory for Quality Ensurance and Sustainable Use of Dao-di Herbs, National Resource Center for Chinese Materia Medica, China Academy of Chinese Medical Sciences, Beijing 100700, China

## Abstract

The triterpenoid-rich *Panax* spp. possess notable allelopathic capabilities, yet current knowledge lacks comprehensive understanding of the biological mechanisms underlying these inhibitory effects. Here, we show that zingibroside R1 (ZR1), a newly identified allelochemical from *Panax* spp., inhibits the adventitious root growth in ginseng and primary root elongation in *Arabidopsis*. Mechanistic analyses revealed that ZR1 disrupts auxin transport, as evidenced by monitoring DR5::GFP and PINs markers. The effect of ZR1 on auxin transport was further elucidated through synergistic effects observed with the auxin transport inhibitor NPA and in the auxin transport mutants *aux1* and *pin2*. Additionally, ZR1 downregulates key root development regulators, including stem cell maintenance regulators PLT1/2, WOX5, SHR, and SCR. Transcriptomic and biochemical data indicate that ZR1 triggers an ROS response in plants, which disrupts ROS scavenging—via altered peroxidase activities and reduced glutathione/oxidized glutathione balance—thereby impairing detoxification function and resulting in root shortening by regulation of auxin distribution and stem cell gene expression. Our results establish a mechanistic framework for understanding how ginsenosides mediate allelopathic effects on root development.

## Introduction

Following germination, plants establish themselves in fixed locations, where they encounter a range of biotic and abiotic factors. To thrive, plants have evolved sophisticated response systems to outcompete neighboring plants and defend against herbivores and pathogens [1, 2]. They achieve this through mechanisms, such as competition for resources, allelopathy, or a combination of both [3, 4]. To date, a wide array of allelochemicals has been identified and studied across various plant species; these include phenolics, flavonoids, coumarins, quinones, terpenoids, alkaloids, and nitrogen-containing chemicals, among others [5, 6]. Triterpenoids act as signaling molecules and allelochemicals, playing essential roles in regulating key plant biological processes, including defense mechanisms, rhizosphere microorganism interactions, autotoxicity, and seed germination [7, 8]. For instance, codonopilate A and taraxeryl acid, exuded by the roots of *Codonopsis pilosula*, induce the accumulation of reactive oxygen species (ROS) in root tips, ultimately triggering autotoxicity [9, 10]; betulinic acid, oleanolic acid, and ursolic acid, compounds isolated from *Alstonia scholaris*, exhibit inhibitory effects on the radicle growth of *Bidens pilosa* and *Lactuca sativa* [11].

The genus *Panax*, recognized for its significant allelopathic properties, encompasses a variety of renowned medicinal herbs, including *Panax ginseng*, *Panax notoginseng*, *Panax japonicus*, and *Panax quinquefolius* [12]. Ginsenosides, the triterpenoid glycosides identified in *Panax* spp., have been recognized as allelochemicals and serve as the primary active constituents, exhibiting notable anti-fatigue, anti-diabetic, and anti-tumor properties [12, 13]. The allelopathic effects of ginsenosides vary significantly depending on their chemical types. The hairy roots of ginseng exhibited significantly greater cell apoptosis when exposed to low-polarity ginsenoside components compared to high-polarity ones [14]. Some rare ginsenosides inflicted more severe damage to ginseng hairy root cells and exhibited a more pronounced inhibition on the growth of ginseng hairy roots [15]. The ginsenosides isolated from the soil of consecutively cultivated *Panax* species similarly demonstrate allelopathic properties. The *n*-butanol extract derived from the soil of continuously cropped ginseng exhibited significant autotoxic activity on both seeds and seedlings of cucumber [16]. Ursane-type triterpenoid acids isolated from the soil of 3-year-old *P. notoginseng* cultivation inhibited the elongation of *Arabidopsis* root tip cells, induced cell death in *Arabidopsis* root tips, caused mortality in *P. notoginseng* root cells, and stimulated the growth of *P. notoginseng* pathogens [17]. The ginsenosides R1, Rg1, Re, Rg2, and Rd, identified in the roots and consecutively cultivated soil of *P. notoginseng*, exhibited autotoxic effects on seedling emergence, growth, and root cell vitality [18]. The allelopathic mechanism of ginsenosides may be associated with the accumulation of ROS and endogenous phytohormones, including jasmonic acid (JA), abscisic acid (ABA), and salicylic acid (SA) [12, 15]. Zingibroside R1 (ZR1), a rare oleanane-type ginsenoside predominantly found in aged and wild *Panax* species (e.g., *P. ginseng* [19], *P. quinquefolius* [19], *P. notoginseng* [20], *P. japonicus* var. *major* [21], *Panax zingiberensis* [22], *P. japonicus* [23]), possesses various bioactivities including antifungal [20], antitumor [24], anti-aging [25], and hepatoprotective [26] properties. Nevertheless, its potential role as a novel allelochemical and the associated molecular mechanisms have yet to be elucidated.

Herein, by screening 12 ginsenosides in both ginseng adventitious roots and *Arabidopsis* roots, we identified ZR1 as exhibiting significant allelopathic effects through modulating auxin transport and root stem cell processes. RNA-seq of treated *Arabidopsis* roots revealed ZR1 might modulate oxidative response pathway to mediate auxin distribution and stem cell genes. This study provides crucial insights for future investigations into the molecular mechanisms by which ginsenosides regulate root development through allelopathic interactions.

## Results

### The allelopathic effects of ginsenosides on root system development both in ginseng and *Arabidopsis*

To investigate the allelopathic effects of ginsenosides on root growth, ginseng adventitious roots were treated with twelve different triterpenoids reported in *Panax* genus, including ZR1, 20(R)-Notoginsenoside R2, Notoginsenoside R1, Araloside A, Calenduloside E, 20(S)-Notoginsenoside R2, Chikusetsusaponin IVa, Ginsenoside Rb2, Ginsenoside Rb3, Ginsenoside Rf, Ginsenoside Ro, and Ginsenoside Rd (Fig. S1). A comparative analysis of the allelopathic impacts exerted by 12 distinct ginsenosides on root morphology revealed that compounds 20(S)-Notoginsenoside R2, ZR1, and Ginsenoside Rf demonstrated pronounced allelopathic properties, significantly inhibiting the growth of ginseng adventitious roots (Fig. S1). To quantify ZR1's dose-dependent effects on ginseng root morphogenesis, we implemented a sterile adventitious root culture system with four conditions: mock control (0 μM ZR1) plus three ZR1 concentrations (20, 50, 100 μM), maintaining strict environmental controls (Fig. 1A). Root density measurements showed that 100 μM ZR1-treated samples exhibited 65.9% and 43.7% reductions relative to control at 30 and 45 days, respectively (*P* < 0.05, *P* < 0.01) (Fig. 1B). Under different ZR1 concentrations, 50and 100 μM ZR1 treatment significantly decreased the root apical meristem (RAM) length of *P. ginseng* adventitious roots after 45 days compared to the control (*P* < 0.05) (Fig. 1C).

**Figure 1:**
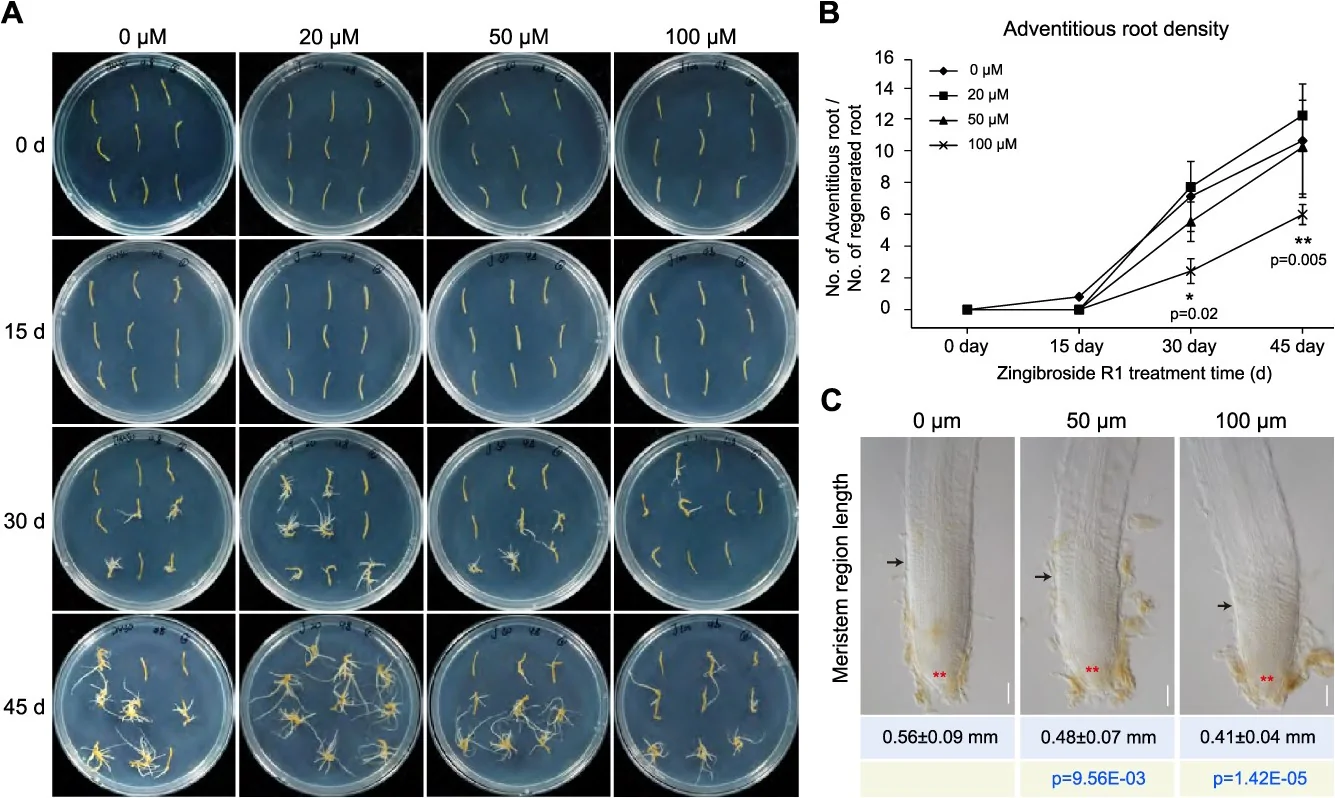
Integrative analysis of morphological alterations in *P. ginseng* adventitious roots following ZR1 treatment. (A) Phenotypic modulation of ginseng adventitious roots in response to 0 μM (Mock), 20, 50, and 100 μM ZR1 treatment. (B) Temporal dynamics of adventitious root density under differential concentrations (0 μM [Mock], 20, 50, and 100 μM) of ZR1 treatment (*n* = 3). Adventitious root density = total number of regenerated adventitious roots/number of roots with regenerated adventitious roots per plate. (C) Root apex architecture in ginseng adventitious roots visualized by DIC microscopy following 45-day treatment with 0 μM (Mock) and 50 and 100 μM of ZR1. The arrowhead marks the boundary between the meristematic zone and the elongation zone. The asterisk marks the QC. Black numbers indicate the mean meristem zone length ± variance. Blue numbers indicate the significance level (*P* value) for differences in meristem zone length between ZR1-treatment and control groups. Scale bar = 50 μm (*n* = 15).

Additionally, *Arabidopsis thaliana* Col-0 seedlings were also treated with the above triterpenoids (Fig. S2). A comparative analysis of the allelopathic impacts exerted by 12 distinct ginsenosides on root morphology revealed that compounds Chikusetsusaponin IVa, ZR1, and Calenduloside E demonstrated pronounced allelopathic properties, significantly inhibiting the elongation of primary roots (Figs S2 and S3). ZR1 exhibited the most substantial dose-dependent inhibition of root morphogenesis among the above evaluated compounds. Significant growth suppression of primary root growth was detected at 10 μM (*P* < 0.05), with complete growth arrest and distinct necrotic lesions at the RAM occurring at concentrations of 50 μM (Fig. S3D–F). To systematically characterize the dose–response effect of ZR1 at subthreshold concentrations, we performed comprehensive phenotypic profiling of *Arabidopsis* root architecture under 5, 7.5, and 10 μM treatments (Fig. 2A and B). At 5 μM, ZR1 treatment resulted in no significant change in root elongation compared to the control (0 μM), but significant inhibition was observed at concentrations of 7.5 and 10 μM, especially after 2 days of treatment (Fig. 2A and B). Two time points (2 and 4 days post-treatment) were selected corresponding to the initial and significant morphological alterations in root meristem phenotypes induced by ZR1 (Fig. 2C). Quantitative analysis of 2-day ZR1 treatments revealed a significant dose-responsive decline in both the root meristem region length and the elongation region length in *Arabidopsis* roots (Fig. 2D and E). The 10-μM treatment group showed the most substantial morphological alterations, with a 45.9% reduction in meristem region length and a 66.1% decrease in elongation region length compared to controls (Fig. 2D and E). Similarly, 4-day treatment with ZR1 caused marked reduction in both root meristem region length and elongation region length (Fig. 2F–H). These collective findings show that ZR1-mediated inhibition of primary root growth occurs through its suppressive effects on both meristematic zone elongation and elongation region length.

**Figure 2:**
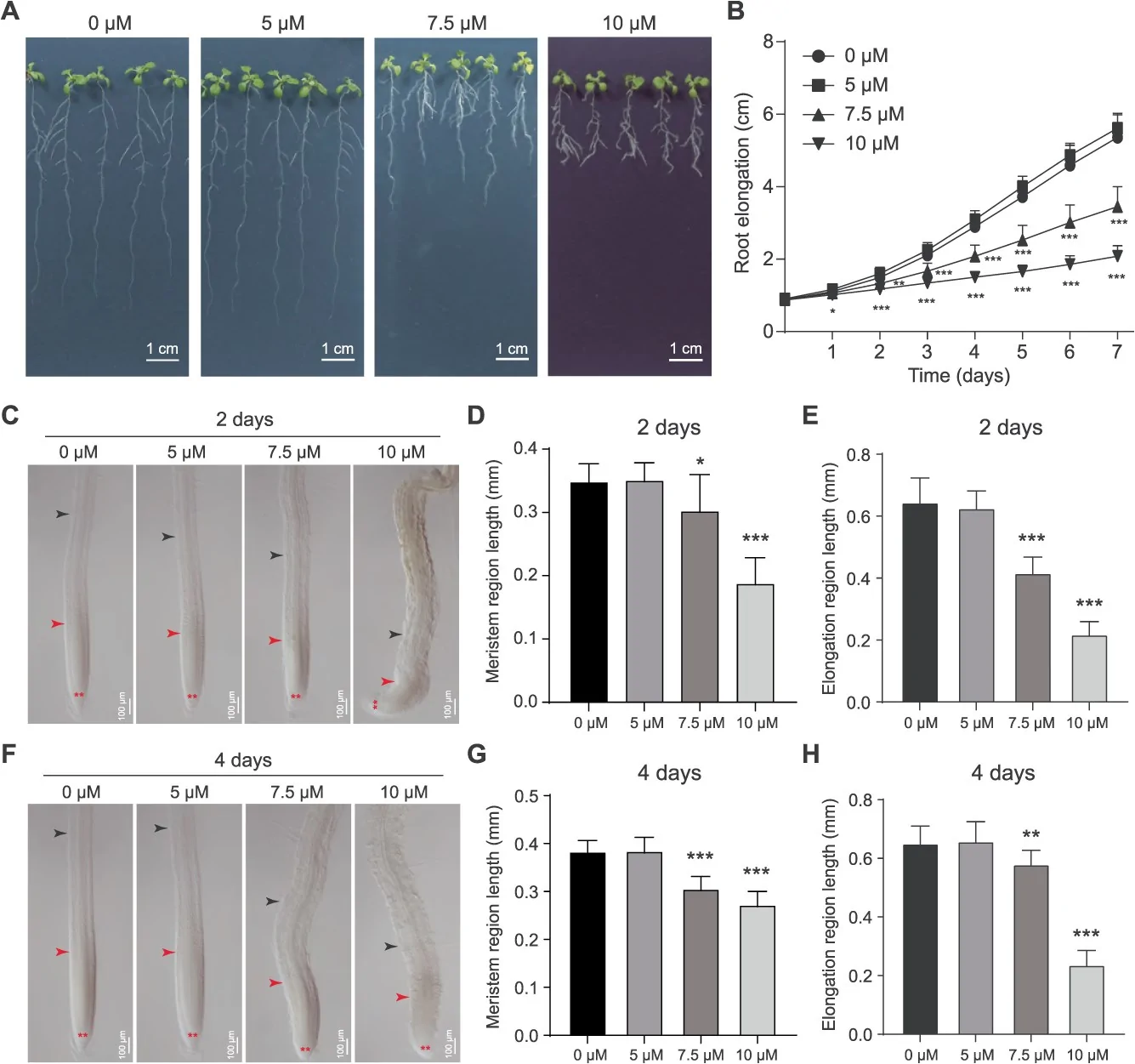
Functional characterization of ZR1-mediated suppression of root system architecture and apical meristem activity in *A. thaliana*. (A) Phenotypic images of *A. thaliana* roots treated with 0, 5, 7.5, and 10 μM of ZR1. (B) Concentration-dependent alterations in root elongation of *A. thaliana* after 7-day exposure to ZR1. (C) Longitudinal view of an RAM of *A. thaliana* with 2-day treatment of 0, 5, and 10 μM of ZR1. The red asterisk marks the QC. The red arrowhead marks the boundary between the meristematic zone and the elongation zone. The black arrowhead marks the boundary between the elongation zone and the maturation zone. Scale bar = 100 μm. (D) Quantitative analysis of meristematic region length in *A. thaliana* root tips following 2-day exposure to ZR1 at concentrations of 0, 5, and 10 μM. (E) Quantitative analysis of meristematic cortex cell number in *A. thaliana* root tips following 2-day exposure to ZR1 at concentrations of 0, 5, and 10 μM. (F) Longitudinal view of a RAM of *A. thaliana* with 4-day treatment of 0, 5, and 10 μM of ZR1. The red asterisk marks the QC. The red arrowhead marks the boundary between the meristematic zone and the elongation zone. The black arrowhead marks the boundary between the elongation zone and the maturation zone. Scale bar = 100 μm. (G) Quantitative analysis of meristematic region length in *A. thaliana* root tips following 4-day exposure to ZR1 at concentrations of 0, 5, and 10 μM. (H) Quantitative analysis of meristematic cortex cell number in *A. thaliana* root tips following 4-day exposure to ZR1 at concentrations of 0, 5, and 10 μM.

### The effects of ZR1 on auxin homeostasis and distribution in *Arabidopsis*

Building upon the observations that ZR1 affects root meristem activity, we further focused on elucidating the molecular mechanisms underlying ZR1's allelopathic effects, with particular emphasis on its mode of action at the cellular level and its impact on key physiological processes.

Given the important role of local auxin distribution and transport in root meristem activity in *Arabidopsis* [27, 28], we assessed whether ZR1 influences auxin pathway. First, we measured IAA concentrations following 7 days of ZR1 treatment, revealing a significant reduction in endogenous IAA levels specifically at 10 μM ZR1 (Fig. S4). To further assess auxin accumulation in root tips, the DR5::GFP reporter was employed [29]. Following a 2-day ZR1 treatment, DR5::GFP signals remained unchanged (Fig. 3A and B), while DR5::GFP fluorescence showed significant reduction specifically at 10-μM concentration after 4 days of ZR1 treatment (Fig. 3C and D). These observations suggest that ZR1 treatment could affect auxin accumulation. To elucidate the molecular mechanisms underlying these effects on auxin distribution, we focused our analysis on PIN-FORMED (PIN) transporters—key regulators of polar auxin transport in plants [30]. After a 2-day exposure to ZR1, PIN1\2\4 exhibited significantly reduced fluorescence intensity specifically at 10 μM (Fig. 3E, F, I, J, M, and  N). After a 4-day treatment with ZR1, only PIN1 exhibited a significant decrease in fluorescence intensity at 10-μM concentration (Fig. 3G and H), while other PIN family members showed no notable changes (Fig. 3K, L, O, and  P). Taken together, the expression of PINs family members exhibited reduction upon the treatment of ZR1.

**Figure 3:**
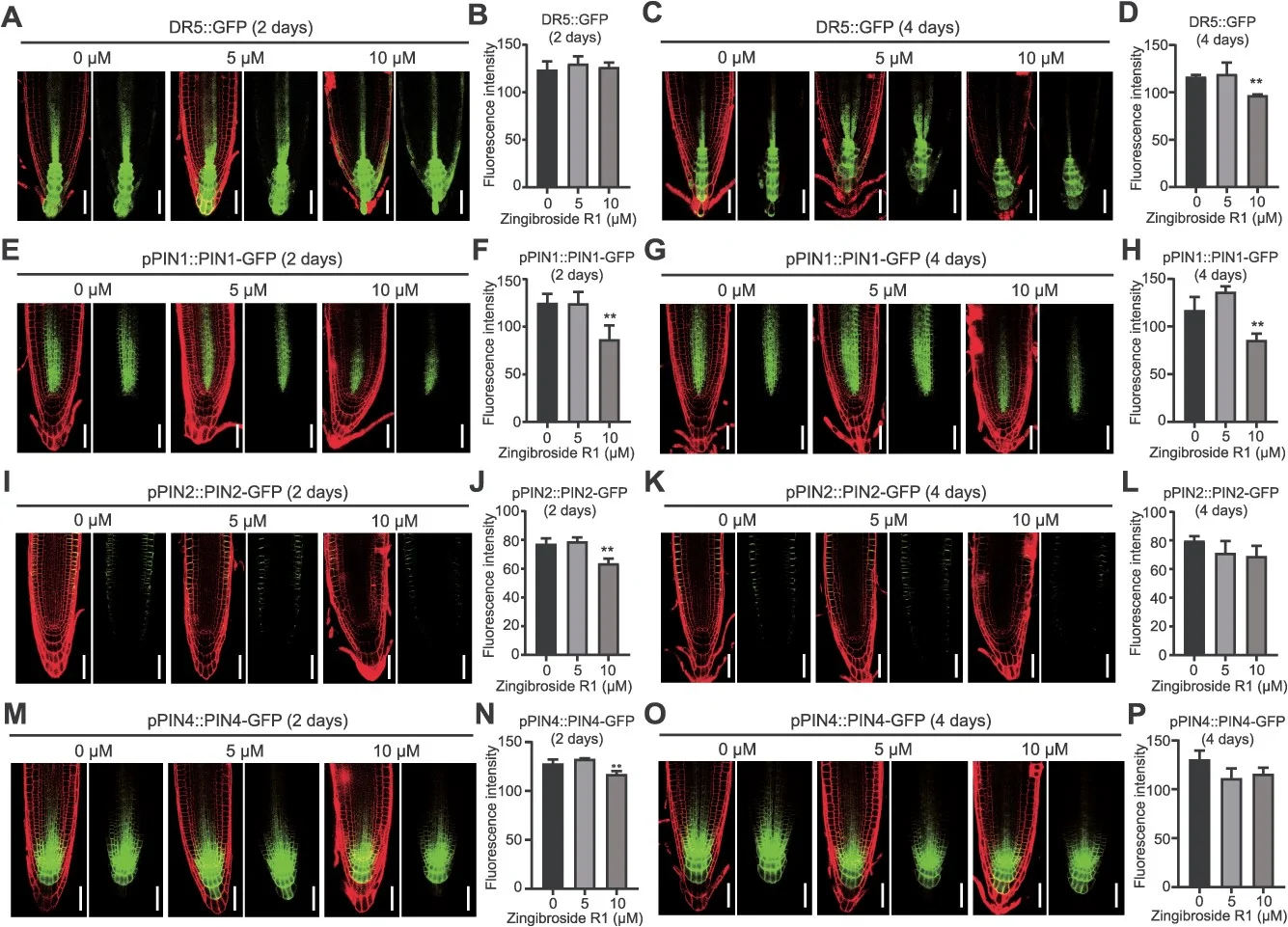
ZR1 effects on expression dynamics of auxin reporter and auxin transport markers. (A and B) Fluorescence intensity of auxin signaling sensor DR5::GFP after 2 days of ZR1 treatment (*n* = 3). Bar = 50 μm. (C and D) Fluorescence intensity of auxin signaling sensor DR5::GFP after 4 days of ZR1 treatment (*n* = 3). Bar = 50 μm. (E and F) Expression of *PIN1::PIN1-GFP* auxin exporter marker following 2-day treatment with ZR1 treatment (*n* = 3). Bar = 50 μm. (G, H) Expression of *PIN1::PIN1-GFP* auxin exporter marker following 4-day treatment with ZR1 (*n* = 3). Bar = 50 μm. (I and J) Expression of *PIN2::PIN2-GFP* auxin exporter marker following 2-day treatment with ZR1 (*n* = 3). Bar = 50 μm. (K and L) Expression of the *PIN2::PIN2-GFP* auxin exporter marker following 4-day treatment with ZR1 treatment (*n* = 3). Bar = 50 μm. (M and N) Expression of *PIN4::PIN4-GFP* auxin exporter marker following 2-day treatment with ZR1 treatment (*n* = 3). Bar = 50 μm. (O and P) Expression of *PIN4::PIN4-GFP* auxin exporter marker following 4-day treatment with ZR1 treatment. *n* = 3. Bar = 50 μm.

To further elucidate the mechanistic interactions, we performed combinatorial pharmacological treatments using: (i) the auxin transport inhibitor NPA (1-N-naphthylphthalamic acid) with ZR1 (Fig. 4), and (ii) the auxin biosynthesis inhibitor KYN (L-kynurenine) [31] with ZR1 (Fig. 5A and B). Compared to individual treatments, combined application of ZR1 and NPA at varying concentrations resulted in more pronounced inhibition of *Arabidopsis* root elongation (Fig. 4A and B), while simultaneously expanding the root meristem zone area (Fig. 4C and D). Moreover, the combined treatment of NPA with ZR1 strongly suppress the lateral root formation, suggesting that ZR1-induced lateral root formation is required on auxin polar transport (Table S1). Compared to ZR1 treatment alone, the combined application of ZR1 and KYN significantly reduced root length, with the most pronounced effect observed at 1.5 μM KYN (Fig. 5A and B). To assess ZR1's effect on auxin transport, we treated the *aux1* and *pin2* mutants with ZR1 and found that it exacerbated their agravitropic coiling and root shortening phenotypes (Fig. S5). To investigate whether ZR1 directly interacts with components of the IAA signaling pathway, we performed molecular docking and molecular dynamics (MD) simulations of ZR1 with key IAA signaling molecules, including TIR1, IAA7, ARF7, and ARF19. Molecular docking revealed that ZR1 binds to TIR1, IAA7, ARF7, and ARF19 with binding affinities of −8.4, −8.1, −7.2, and − 8.4 kcal/mol, respectively (Figs S6–S9). MD simulations further showed that ZR1 forms a relatively stable complex with TIR1 and ARF7 over 100 ns (Fig. 5C and D; Fig. S10), while its interactions with IAA7 and ARF19 appeared unstable during the same period (Figs S11 and S12), which suggest that ZR1 might bind stably to TIR1 and ARF7. Additionally, molecular docking revealed that IAA binds to TIR1 and ARF7 with binding affinities of −6.4 and − 5.5 kcal/mol, respectively, suggesting that ZR1 may bind more readily to these proteins than IAA (Figs S13 and S14). Further analysis of the molecular docking binding modes revealed that both ZR1 and IAA form abundant van der Waals and conventional hydrogen bond interactions with key residues within the binding cavities of the target proteins TIR and ARF7 (Figs S6, S8, S13, and  S14). Specifically, IAA binding to TIR additionally involves Pi-Cation (ARG-509) and Pi-Alkyl (LYS-74, LYS-485, PRO-77) interactions (Figs S13), whereas IAA binding to ARF7 further includes Pi-Sigma (PRO-1081) and Pi-Alkyl (LEU-1099) interactions (Fig. S14). Based on transcriptomic data, we next examined the expression of *TIR1* and *ARF7* in response to ZR1 treatment. As ZR1 concentration increased, *TIR1* expression showed a significant upward trend, whereas *ARF7* expression initially decreased before returning to levels comparable to the control (Fig. S15). These findings indicate that ZR1 might exert a regulatory effect on *TIR1* expression. Integrating the molecular simulation and expression data, we hypothesize that ZR1 may inhibit the auxin receptor TIR1, leading to a compensatory upregulation of *TIR1* expression to maintain normal IAA signaling and mitigate ZR1’s allelopathic effects.

**Figure 4:**
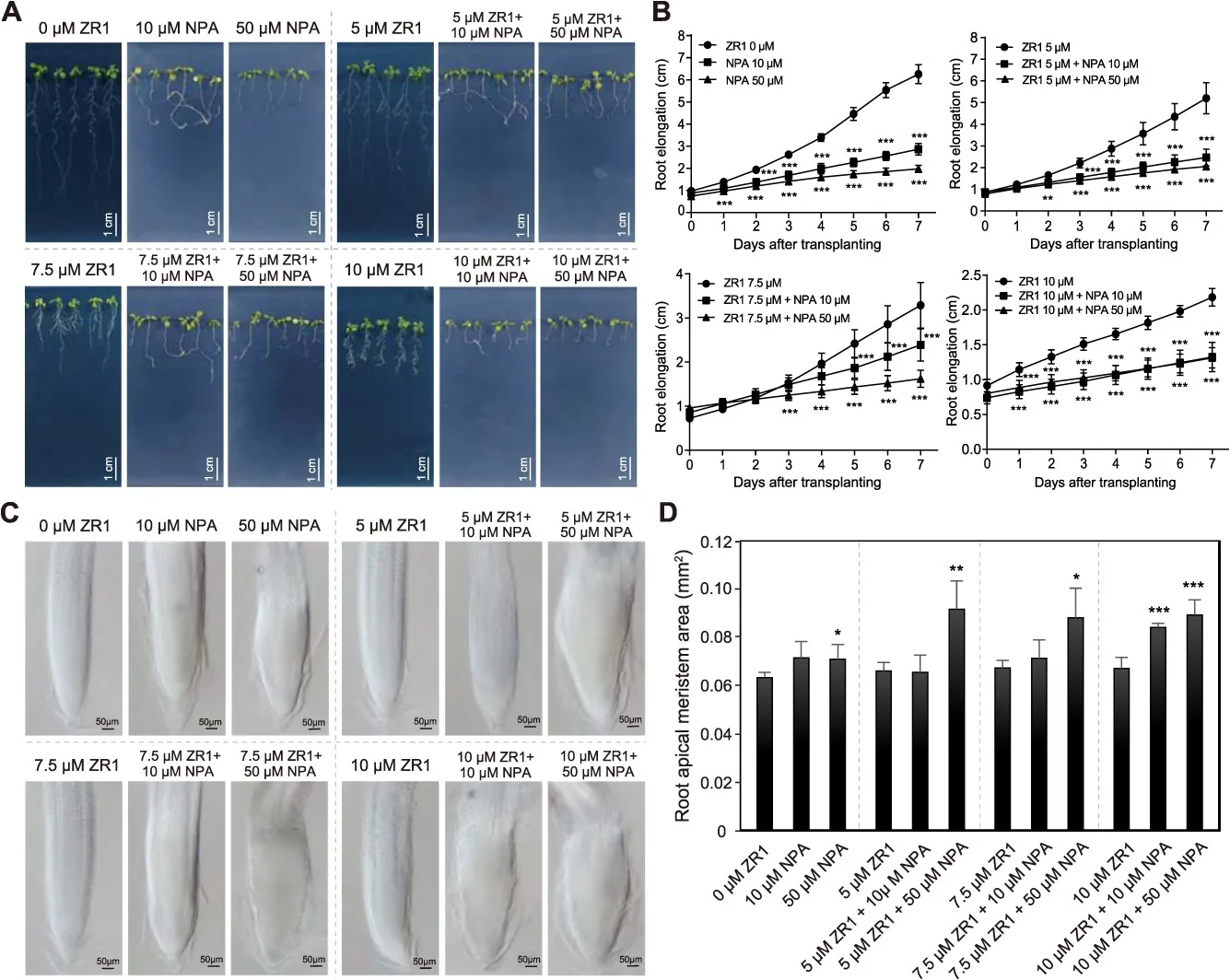
Synergistic effects of ZR1 co-treatment with the polar auxin transport inhibitor N-1-naphthylphthalamic acid (NPA). (A) Phenotypic modifications of root system development in *A. thaliana* following treatment with ZR1 and NPA. (B) Concentration-dependent alterations in root elongation of *A. thaliana* after 7-day exposure to ZR1 and NPA. ^***^P < 0.001; ^**^P < 0.01. (C) Root apex architecture in Col-0 seedlings visualized by DIC microscopy following 7-day treatment with various concentration of ZR1 and NPA. Scale bar = 50 μm (*n* = 5). (D) Quantitative analysis of RAM size alterations in response to 7-day combinatorial treatment with gradient concentrations of ZR1 and NPA (*n* = 5). ^***^P < 0.001; ^**^P < 0.01; ^*^P < 0.05.

**Figure 5:**
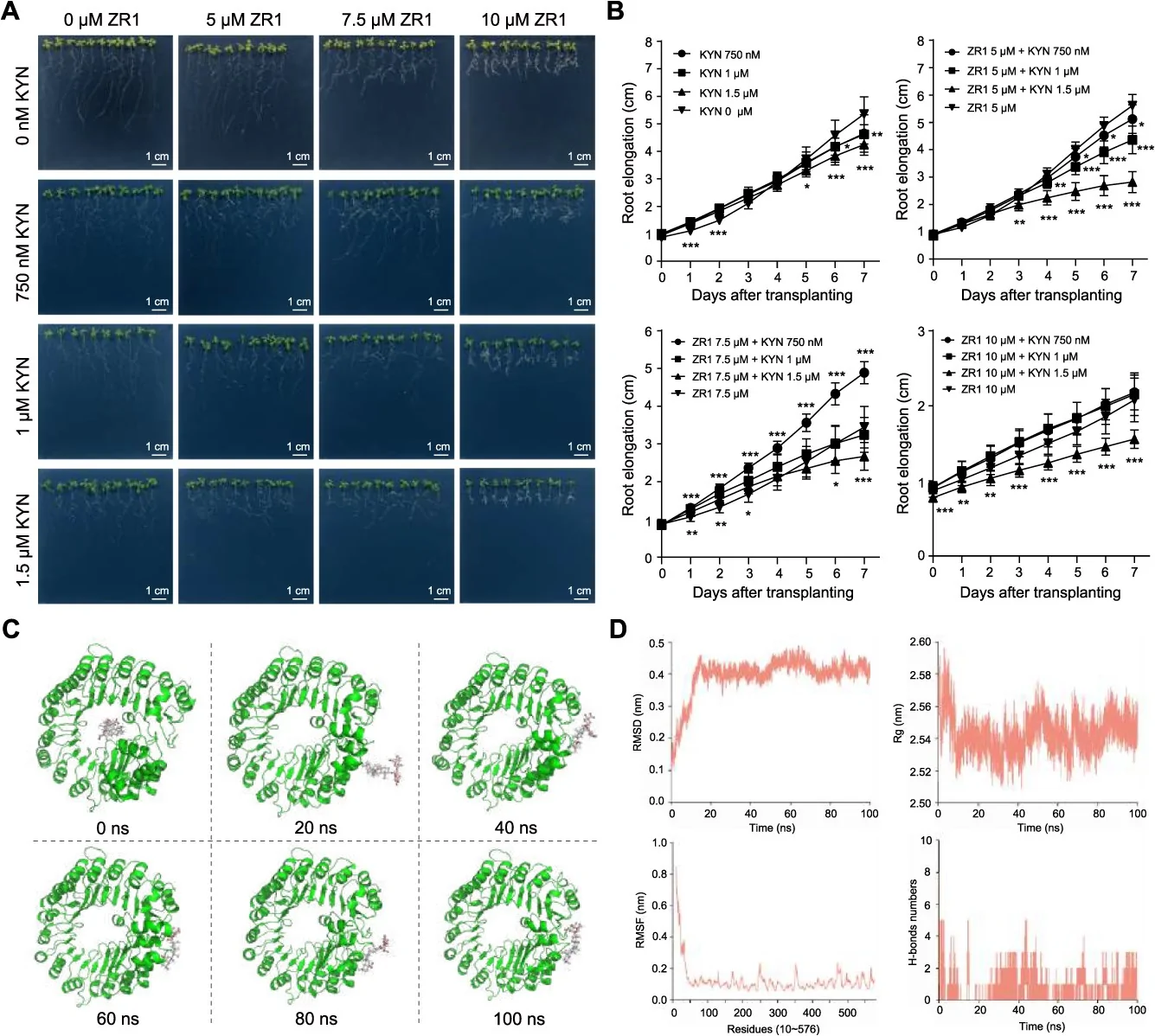
Unveiling the link between ZR1 and auxin synthesis and signal transduction. (A) Combined effects of ZR1 and the auxin biosynthesis inhibitor KYN on *Arabidopsis* root phenotype. (B) Concentration-dependent alterations in root elongation of *A. thaliana* after 7-day exposure to ZR1 and KYN (*n* = 10). ^***^P < 0.001; ^**^P < 0.01; ^*^P < 0.05. (C) Conformational dynamics of the ZR1–TIR1 ligand–receptor complex during 100 ns MD simulation. (D) MD simulation results of the ZR1–TIR1 ligand–receptor complex, including the RMSD of the ZR1–TIR1 complex, Rg of the ZR1–TIR1 complex, RMSF of the ZR1–TIR1 complex, and the number of hydrogen bonds of the ZR1–TIR1 complex.

Taken together, our imaging, phenotypic, and pharmacological analyses indicate that root growth-inhibitory effects of ZR1 are mediated predominantly via effect on auxin pathway.

### The effects of ZR1 on expression of root stem cell regulators and root regenerative capacity

In view of the fundamental role played by transcription factors in the regulation of root stem cells and root development processes, we evaluated the effect of ZR1 on activity of PLT, WOX5 and SCR/SHR transcription factors—master regulators of root stem cell maintenance. Exposure to 10 μM ZR1 for 2 and 4 days led to a significant reduction in the fluorescence intensity of pSCR::SCR-GFP and pSHR::SHR-GFP in *Arabidopsis* root (Fig. 6A–H), whereas treatment with 5 μM ZR1 for 2 days only significantly decreased the fluorescence of pSCR::SCR-GFP (Fig. 6A-H). Treatment with 10 μM ZR1 for either 2 or 4 days also significantly reduced the fluorescence intensity of both pPLT1::PLT1-YFP and pPLT2::PLT2-GFP (Fig. 6I–P). Additionally, after 2 days of treatment with 5 μM ZR1, the fluorescence intensity of pPLT1::PLT1-YFP showed a significant decrease, while that of pPLT2::PLT2-GFP exhibited a marked increase (Fig. 6I–P). After 4-day exposure to ZR1, pWOX5::GFP exhibited significantly induced fluorescence intensity specifically at 5 and 10 μM (Fig. 6Q–T).

**Figure 6:**
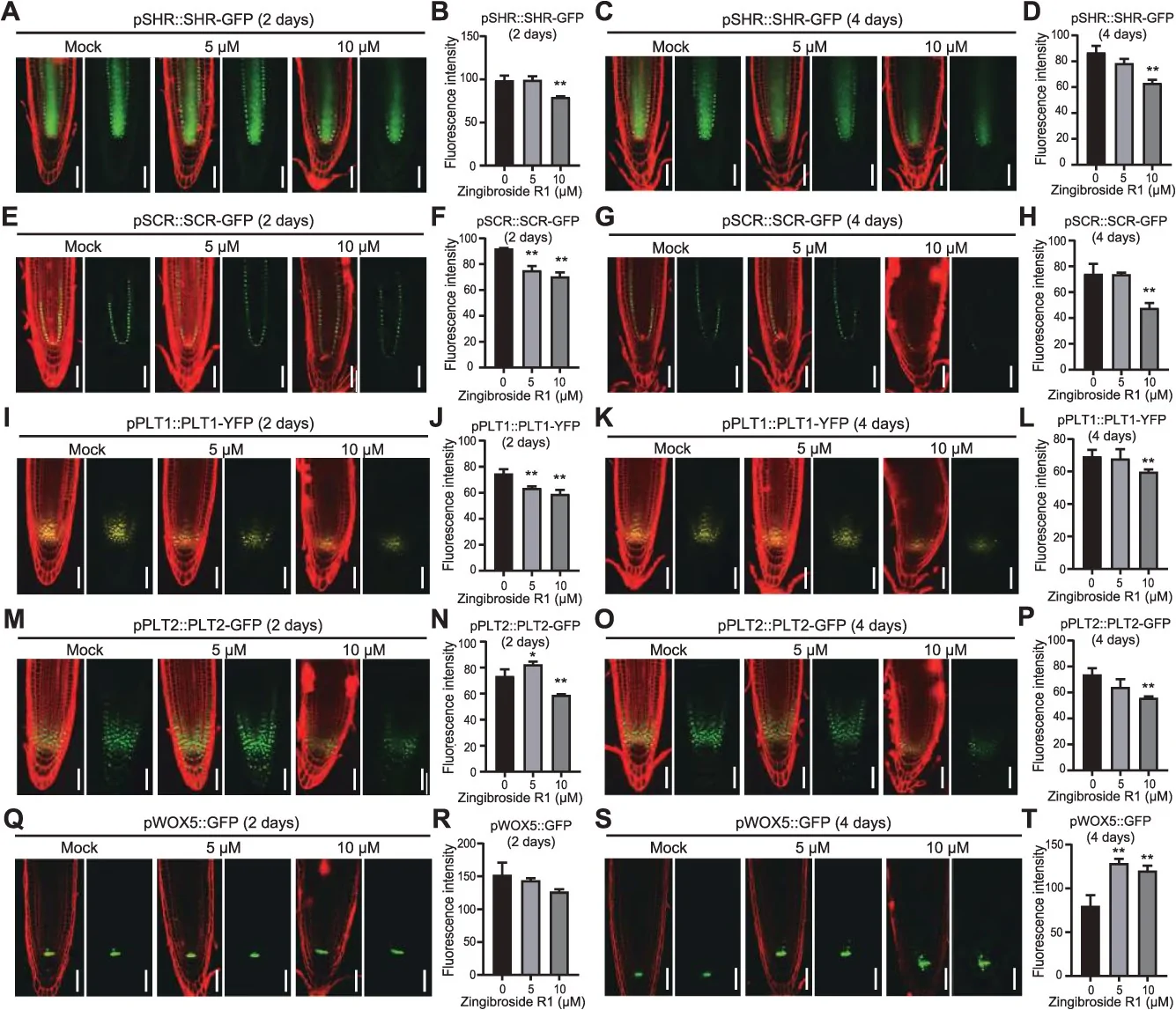
Expression dynamics of root stem cell regulators. (A and B) Fluorescence intensity of *pSHR::SHR-GFP* after 2 days of ZR1 treatment (*n* = 3). Bar = 50 μm. (C and D) Fluorescence intensity of *pSHR::SHR-GFP* after 4 days of ZR1 treatment (*n* = 3). Bar = 50 μm. (E and F) Fluorescence intensity of *pSCR::SCR-GFP* after 2 days of ZR1 treatment (*n* = 3). Bar = 50 μm. (G and H) Fluorescence intensity of *pSCR::SCR-GFP* after 4 days of ZR1 treatment (*n* = 3). Bar = 50 μm. (I and J) Fluorescence intensity of *pPLT1::PLT1-YFP* after 2 days of ZR1 treatment (*n* = 3). Bar = 50 μm. (K and L) Fluorescence intensity of *pPLT1::PLT1-YFP* after 4 days of ZR1 treatment. *n* = 3. Bar = 50 μm. (M and N) Fluorescence intensity of *pPLT2::PLT2-GFP* after 2 days of ZR1 treatment. *n* = 3. Bar = 50 μm. (O and P) Fluorescence intensity of *pPLT2::PLT2-GFP* after 4 days of ZR1 treatment (*n* = 3). Bar = 50 μm. (Q and R) Fluorescence intensity of *pWOX5::GFP* after 2 days of ZR1 treatment (*n* = 3). Bar = 50 μm. (S and T) Fluorescence intensity of *pWOX5::GFP* after 4 days of ZR1 treatment (*n* = 3). Bar = 50 μm.

Given the association between root stem cells and regenerative capacity [32, 33], we evaluated the influence of ZR1 on plant regeneration through the capacity of callus induction experiments (Fig. S16). Following root elongation to 1.0 cm, *Arabidopsis* seedlings were aseptically transferred to callus induction medium (CIM) containing ZR1 at concentrations ranging from 0 to 10 μM (Fig. S16A). However, after 7 days of ZR1 treatment, no significant difference in callus area was observed between the treatment groups and the control group (Fig. S16B and C).

In summary, our results demonstrate that although ZR1 treatment significantly modulates the expression of core root stem cell regulators (PLT1/2, WOX5, and SCR/SHR) explaining effects on root meristem size and root growth, there is no detectable enhancement of regenerative capacity in the callus induction experimental system.

### Transcriptomics reveal role of ZR1 in oxidative response pathway

To systematically investigate the effects of ZR1 on transcriptional networks and signaling pathways in *Arabidopsis* roots, we performed RNA-seq analysis on root tissues treated with 0, 5, and 10 μM ZR1. Each root sample yielded >6.6 Gb of clean reads postfiltering, demonstrating high sequencing accuracy (error rate < 3%). The sequencing data exhibited excellent quality (Q20 > 97%, Q30 > 93%) and stable GC content (44.5–45.5%), validating data integrity. Principal component analysis clearly segregated the samples into three distinct clusters corresponding to control, 5 μM ZR1-treated, and 10 μM ZR1-treated groups (Fig. 7A). The high reproducibility of our data was evidenced by Pearson correlation coefficients (*R*^2^ > 0.98) among biological replicates within each treatment group (Fig. 7B).

**Figure 7:**
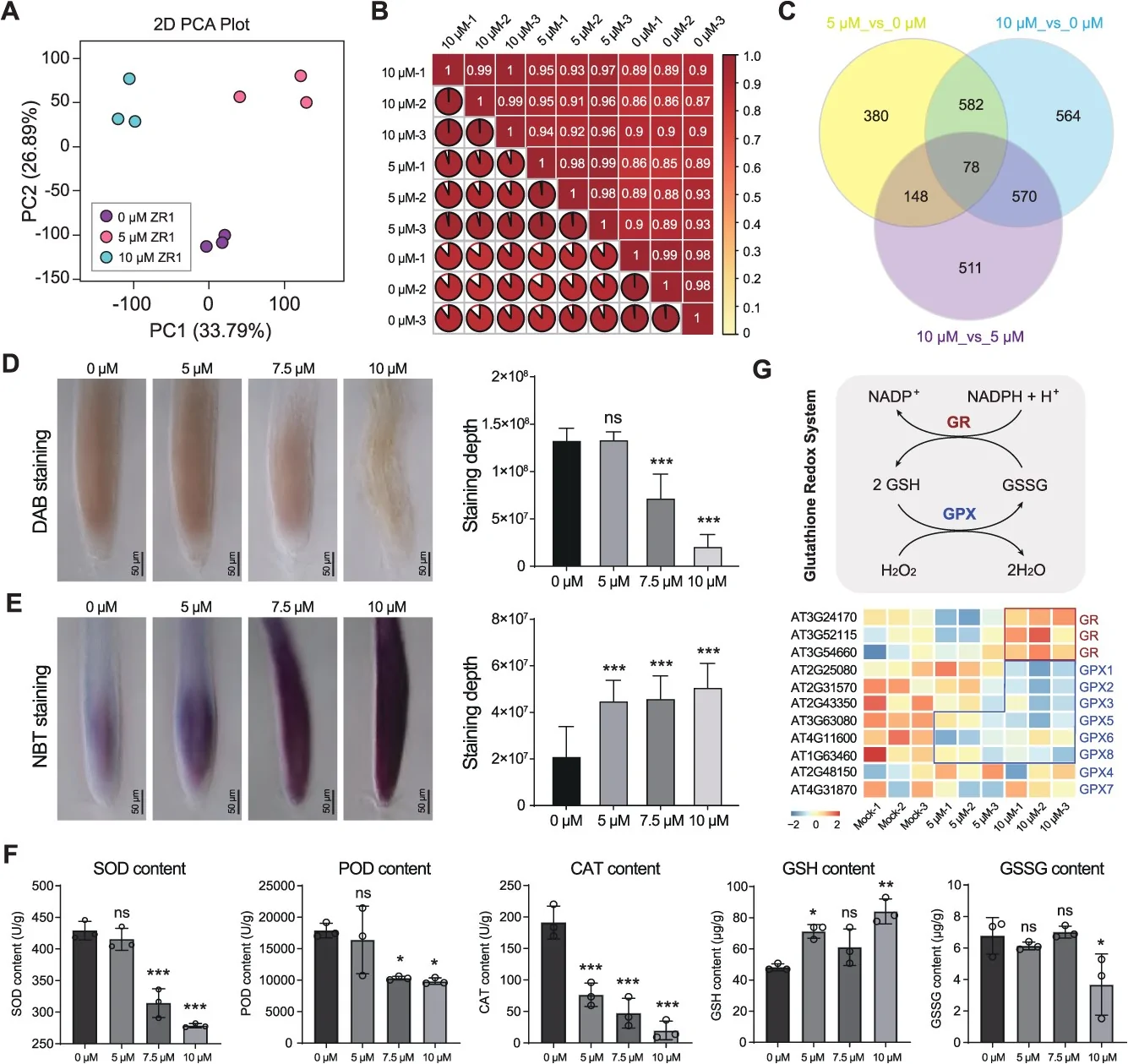
Transcriptomic analysis of *Arabidopsis* root and ginseng adventitious root after ZR1 treatment. (A) PCA analysis of *Arabidopsis* root under 7 days of ZR1 treatment. Mock, the control group without ZR1 treatment; 5 μM ZR1, 5 μM ZR1 treated group; 10 μM ZR1, 10 μM ZR1 treated group (*n* = 3). (B) The square values of Pearson’s correlation coefficients (R [2]) between samples. (C) Comparative analysis of DEGs across ZR1 treatment groups using Venn diagram visualization. (D) DAB staining after ZR1 treatment (*n* = 3). (E) NBT staining after ZR1 treatment (*n* = 3). (F) Effect of ZR1 treatment on the levels of SOD, POD, CAT, and the contents of GSH and GSSG (*n* = 3). (G) Expression analysis of key genes *GR* and *GPX* in the glutathione redox system after ZR1 treatment (*n* = 3).

To compare the overlap of differentially expressed genes (DEGs) across treatment groups, Venn diagrams and Volcano plots were constructed to visualize shared and unique DEGs between 5 μM ZR1-treated group vs control group, 10 μM ZR1-treated group vs control group, and 10 μM ZR1-treated group vs 5 μM ZR1-treated group (Figs 7C and S17). Venn diagram analysis demonstrated 78 core DEGs consistently regulated in all three comparisons, and a significant overlap of 660 DEGs between the two ZR1-treated groups when each was compared to control (Fig. 7C). To elucidate the biological processes associated with these DEGs, we performed gene ontology (GO) enrichment analysis to identify significantly overrepresented functional categories. Comparative GO enrichment analysis revealed that the shared DEGs between both ZR1-treated groups and controls were significantly enriched in oxidative responses (FDR < 0.05) (Fig. S18).

To assess ROS accumulation in the RAM, 3,3′-diaminobenzidine (DAB) and nitro blue tetrazolium (NBT) staining were performed following ZR1 treatment. Our results demonstrated that with increasing concentrations of ZR1 treatment, DAB staining gradually decreased, while NBT staining became progressively deeper, which indicates that ZR1 treatment increases the concentration of superoxide anions (O_2_^−^) in the root tips, while reducing the concentration of hydrogen peroxide (H_2_O_2_) (Fig. 7D and E). To elucidate the cellular antioxidant response following ZR1 treatment, the activities of SOD, POD, and CAT, as well as the levels of reduced glutathione (GSH) and oxidized glutathione (GSSG), were determined. The results showed that decreased activities of the key ROS-scavenging enzymes SOD, CAT, and POD, which suggests an impairment in their ability to detoxify ROS (Fig. 7F). In plant oxidative responses, the GSH/GSSG balance serves as a key detoxification pathway. Following 10-μM ZR1 treatment, the significant increase in GSH and decrease in GSSG resulted in an elevated GSH/GSSG ratio (Fig. 7F), mirroring the *GR* and *GPX* expression patterns (Fig. 7G). These findings suggest that ZR1 treatment impairs ROS scavenging in root tips by inactivating SOD, CAT, and POD, thereby blocking superoxide conversion to H₂O₂; this renders the GPX-mediated GSH utilization unnecessary, resulting in an increased GSH/GSSG ratio.

## Discussion

Allelochemicals, a class of secondary plant metabolites, predominantly induce crop physiological disorders although occasionally exhibit growth-promoting effects [7]. Triterpenoids derived from *Panax* spp. are typically considered allelopathic agents contributing to soil sickness in continuous cropping systems [12]. However, comprehensive evaluation of the allelopathic properties of *Panax*-derived triterpenoids remains inadequate, and the underlying molecular mechanisms governing these allelopathic interactions are yet to be fully elucidated. In this study, we characterized ZR1 as a novel allelochemical from *Panax* species, which inhibits the adventitious root growth in ginseng and suppresses primary root elongation in *Arabidopsis* (Figs 1 and 2).

To elucidate the underlying mechanism of the allelopathic ZR1 effect on root systems, we employed *Arabidopsis* reporter lines monitoring auxin distribution (DR5::GFP), polar auxin transport (pPIN1::PIN1-GFP, pPIN2::PIN2-GFP, pPIN4::PIN4-GFP), and stem cell regulation (pSHR::SHR-GFP, pSCR::SCR-GFP, pPLT1::PLT1-YFP, pPLT2::PLT2-GFP, pWOX5::GFP) activity. Our analyses demonstrated that ZR1 reduces auxin accumulation in the root tips, disrupts polar auxin transport, and downregulates key stem cell regulatory genes (Figs 3−6), providing a plausible explanation for the root meristem and root growth regulation by ZR1. Consistent patterns of effects were observed also in the *P. ginseng* adventitious root system (Fig. 1). Furthermore, molecular docking and dynamics simulations suggest that ZR1 may stably bind to TIR1 and ARF7, but this finding requires further validation through future biochemical and biophysical experiments. Collectively, these observations imply that ZR1 might modulate root development through its coordinated effects on auxin distribution/transport and subsequent regulation of stem cell-related genes.

*Arabidopsis* transcriptomic analysis further revealed that ZR1 treatment significantly perturbs oxidative response pathway (Fig. 7). As the central antioxidant system, the GSH/GSSG redox cycle scavenges ROS to maintain cellular oxidative balance and involve in plant regeneration [34, 35]. To counteract ZR1 effects, the glutathione redox cycle undergoes substantial transcriptional reprogramming, leading to GSH accumulation in *Arabidopsis*. GSH, a potent nonenzymatic antioxidant, participates in the regulation of diverse biological processes including growth, development, cell cycle progression, gene expression, and protein activity through modulation of cellular redox status [36, 37]. Crucially, GSH levels regulate auxin polar transport by modulating PIN protein stability, while its metabolic coupling with the pentose phosphate pathway via NADPH regeneration sustains ATP production under hypoxia [38, 39], thereby influencing root aerenchyma formation. Similar transcriptomic analysis of ZR1-treated ginseng adventitious roots revealed significant downregulation of key glutathione redox cycle genes, including *GPX* and *GR*, indicating substantial suppression of glutathione pathway activity by ZR1 treatment in ginseng. The inhibition of GSH/GSSG cycling exacerbated oxidative damage in plants [40]. These findings provide novel insights into the allelopathic mechanisms of *P. ginseng*.

In summary, we have identified ZR1 as a novel allelochemical derived from *Panax* species. Our findings demonstrate that ZR1 modulates root development through inhibition of auxin polar transport and subsequent alteration of auxin distribution. Furthermore, ZR1 exerts oxidative stress and autotoxic effects by regulating the GSH/GSSG redox system. Future investigations should systematically examine ZR1's impact on plant redox homeostasis to elucidate its precise mechanism of action.

## Materials and methods

### Plant materials and tissue culture

In this study, *A. thaliana* Col-0 was utilized as the wild-type control. The plants were cultivated under strictly controlled environmental conditions, maintaining a constant temperature of 22°C with a 16-hour light/8-hour dark photoperiod. The solid growth medium consisted of 1/2 Murashige and Skoog (MS) basal salt mixture (Coolaber, China) supplemented with 1% (w/v) sucrose, 1% (w/v) agar (Duchefa Biochemie, The Netherlands), and MES. Furthermore, a comprehensive set of auxin-related and stem cell marker lines was employed in this study, encompassing the following reporter constructs: *DR5::GFP* for auxin response [41], *pPIN1::PIN1-GFP* [42], *pPIN2::PIN2-GFP* [43, 44], *pPIN3::PIN3-GFP* [45], and *pPIN4::PIN4-GFP* [46] for auxin efflux carriers, *pSHR::SHR-GFP* [47] and *pSCR::SCR-GFP* [48] for root stem cell regulators, as well as *pPLT1::PLT1-YFP*, *pPLT2::PLT2-GFP* [49], and *pWOX5::GFP* [43] for stem cell niche markers; *aux1* [50] and *pin2* [51] mutants for auxin transport.

The adventitious root culture system of *P. ginseng* was established following a well-documented protocol [13], with the following standardized procedures: adventitious roots were induced from callus tissues derived from Jilin ginseng (*P. ginseng* C. A. Meyer) roots and maintained in MS medium containing 3% (w/v) sucrose and indole-3-butyric acid (IBA), with incubation under dark conditions at 25 ± 1°C. Adventitious roots were aseptically excised from explants and subsequently subcultured in 1/2 MS liquid medium supplemented with 3.0 mg∙L^−1^ indole-3-butyric acid (IBA). The cultures were maintained under continuous darkness at 25 ± 1°C with constant agitation at 100 rpm using an orbital shaker. Subculturing was performed at 3-week intervals to establish stable root cultures. After three consecutive subculture cycles (totaling 12 weeks), vigorously growing adventitious roots with stable growth characteristics were selected for subsequent experimental analyses.

### Chemical treatments

Stock solutions of ZR1 (CAS#80930-74-1), 20(R)-Notoginsenoside R2 (20R-NR2, CAS#948046-15-9), Notoginsenoside R1 (NR1, CAS#80418-24-2), Araloside A (AA, CAS#7518-22-1), 20(S)-Notoginsenoside R2 (20S-NR2, CAS#80418-25-3), Chikusetsusaponin IVa (IVa, CAS#51415-02-2), Ginsenoside Rb2 (Rb2, CAS#11021-13-9), Ginsenoside Rb3 (Rb3, CAS#68406-26-8), Ginsenoside Rf (Rf, CAS#52286-58-5), Ginsenoside Ro (Ro, CAS#34367-04-9), Calenduloside E (CE, CAS#26020-14-4), Ginsenoside Rd (Rd, CAS#52705-93-8), and 1-naphthylphthalamic acid (NPA, CAS#132-66-1) were prepared in DMSO, and indole-3-acetic acid (IAA, CAS#87-51-4) was prepared in 1 N NaOH. The above twelve triterpene saponins were commercially obtained (Beiterenkang, Beijing, China), all with purities exceeding 98%.

For individual treatments, *Arabidopsis* Col-0 seedlings with approximately 1 cm roots were transferred to 1/2 MS medium with varying concentrations of ZR1 (0, 5, 7.5, 10, 50, 100, and 200 μM), 20R-NR2 (0, 100, and 200 μM), NR1 (0, 100, and 200 μM), AA (0, 100, and 200 μM), 20S-NR2 (0, 100, and 200 μM), IVa (0, 10, 50, 100, and 200 μM), Rb2 (0, 100, and 200 μM), Rb3 (0, 100, and 200 μM), Rf (0, 100, and 200 μM), Ro (0, 100, and 200 μM), CE (0, 10, 20, 100, and 200 μM), Rd (0, 100, and 200 μM), NPA (10 and 50 nM), and IAA (10 and 50 nM). For combined treatments, seedlings with approximately 1 cm roots were transferred to 1/2 MS medium containing 10 nM NPA along with 5, 7.5, and 10 μM ZR1; 50 nM NPA along with 5, 7.5, and 10 μM ZR1; 10 nM IAA along with 5, 7.5, and 10 μM ZR1; and 50 nM IAA along with 5, 7.5, and 10 μM ZR1, respectively.

Adventitious roots of ginseng were cut into 1 cm segments and transferred onto 1/2MS solid medium supplemented with 2.5 mg∙L^−1^ IBA and varying concentrations of ZR1 (0, 20, 50, and 100 μM). The cultures were maintained under controlled conditions, and ginseng adventitious root growth efficiency was systematically evaluated by quantifying regeneration rate and root density at 0, 15, 30, and 45 days post-treatment using digital image analysis.

### Root length and meristem size analysis

Following chemical treatments, root images were mounted on transparent slides and subjected to differential interference contrast (DIC) microscopy analysis using a NIKON ECLIPSE microscope. Root developmental zones were defined based on cortical cell morphology: the meristematic zone was measured from the quiescent center (QC) to the first cortical cell that began to elongate (defined as being significantly longer than the preceding cell); the elongation zone extended from the first elongated cell to the emergence of the first visible root hair bulge; and the maturation zone started from the first root hair and extended basipetally (Fig. S19) [52]. RAM characteristics were quantified from DIC images using ImageJ (NIH, USA, http://rsb.info.nih.gov/ij). After setting a 0.1-mm scale, RAM length was measured along the cortical file using the Segmented Line tool, RAM area was calculated within a fixed rectangular region, and meristematic cell number was counted using the multipoint tool.

### Confocal microscopic analysis

Following root elongation to 1 cm in 1/2 MS medium, *A. thaliana* marker line seedlings were aseptically transferred to treatment plates containing 1/2 MS medium supplemented with different concentrations of ZR1. Root tips were stained with 10 mg/L propidium iodide (PI) and imaged using a laser scanning confocal microscope (Olympus FV3000RS) equipped with a solid-state or argon-ion laser (power ≥ 65 mW). The excitation wavelengths were set at 488 nm for GFP, 514 nm for YFP, and 561 nm for PI. Fluorescence intensities of GFP/YFP were quantified using Image J software. For images of various auxin-related and stem cell markers, original files were converted to 8-bit format and thresholded using consistent parameters. With the ‘Dark Background’ option enabled, consistent regions of interest (ROIs) with identical areas were defined for each marker based on the primary fluorescence signal distribution. Fluorescence intensity was subsequently calculated using the ‘analyze to measure’ function. The number of biological replicates for each experimental condition is specified in the corresponding figure legends.

### *Arabidopsis* root regeneration analysis

For callus induction analysis, 7-day-old *A. thaliana* Col-0 seedlings with approximately 1 cm long roots were aseptically transferred to CIM containing 1/2 MS supplemented with 0.5 mg/L 2,4-dichlorophenoxyacetic acid (2,4-D), 0.05 mg/L kinetin (KT), and varying concentrations of ZR1. Following 7 days of incubation under controlled conditions, seedlings were subjected to microscopic examination using DIC optics. Callus formation was quantitatively assessed by measuring callus area using ImageJ software (NIH, USA, http://rsb.info.nih.gov/ij) with standardized protocols. For each treatment group, a minimum of 20 biological replicates were analyzed to determine mean values and standard deviations (SD).

### Transcriptomic analysis

*Arabidopsis* Col-0 seeds were germinated and grown on 1/2 MS media containing varying concentrations of ZR1 (0, 5, and 10 μM) for 10 days under controlled growth conditions. In parallel, ginseng adventitious roots were subjected to ZR1 treatments (0 and 20 μM) for 30 days. For transcriptomic analysis, root samples were collected using sterile double-edged blades, immediately flash-frozen in liquid nitrogen, and stored at −80°C until further processing. Three independent biological replicates were prepared for each treatment condition. Total RNA was extracted from approximately 200 mg of liquid nitrogen-pulverized tissue using TRIzol™ reagent (Thermo Fisher Scientific, Waltham, MA, USA) following the manufacturer's protocol. RNA integrity and concentration were assessed using a NanoDrop 2000 spectrophotometer (Thermo Fisher Scientific, USA). First-strand cDNA synthesis was performed with 0.5 μg of total RNA using a cDNA Synthesis Kit (Takara, Japan), followed by second-strand cDNA synthesis employing DNA polymerase I, dNTPs, and RNase H. The resulting double-stranded cDNA was purified using a PCR extraction kit and processed through end repair, A-tailing, and adapter ligation to construct sequencing libraries. High-throughput sequencing was conducted on the Illumina NovaSeq 6000 platform (Illumina, San Diego, CA, USA). The raw sequencing data have been deposited in the Genome Sequence Archive [53] of the National Genomics Data Center [54] under accession number CRA012700 and are publicly accessible at https://ngdc.cncb.ac.cn/gsa. *De novo* transcriptome assembly was performed using the Trinity software package with default parameters [55].

### Molecular docking and MD simulation

Protein structures for TIR1, ARF7, ARF19, and IAA7 were retrieved from the UniProtKB database (IDs: Q570C0, P93022, Q8RYC8, Q38825; https://www.uniprot.org/). The three-dimensional structures of the above proteins, determined by X-ray crystallography (RCSB ID: 2P1M), were selected for molecular docking. The ligand structures were constructed using Chem3D and optimized with the MMFF94 force field. For predocking processing, AutoDock Tools (ADT 1.5.7) were used to add nonpolar hydrogens and Gasteiger charges to the TIR1, ARF7, ARF19, and IAA7 structure, and the above proteins and ligand PDB files were converted to PDBQT format. Semi-flexible docking was subsequently performed using AutoDock Vina. The docking results were visualized and analyzed using PyMOL 2.5 (open-source, Schrödinger) and Discovery Studio Visualizer (open-source, BIOVIA).

MD simulations were conducted based on the protocol described by Zhao *et al.* [56] with slight modifications. The ligand topology was generated using the Generalized AMBER Force Field (GAFF) via the Antechamber module in AMBER 18 [57]. The protein topology was constructed using the Amber99sb-ildn force field within the GROMACS 19.5 software package (https://manual.gromacs.org/) and subsequently combined with the ligand topology to generate the protein–ligand complex topology. All MD simulations were performed using GROMACS 19.5. The complex was solvated with TIP3P water molecules in a cubic box under periodic boundary conditions, and 70 Na^+^ and 68 Cl^−^ ions were added to neutralize the system charge. Energy minimization was carried out using the steepest descent algorithm until the force reached below 1000.0 kJ/mol/nm. The system was then equilibrated under constant temperature and volume (NVT) for 2 ns, followed by constant temperature and pressure (NPT) for 4 ns, maintaining a temperature of 310.15 K and a pressure of 0.1 MPa. A 100-ns production MD run was subsequently performed, accelerated using an NVIDIA GeForce RTX 3080 GPU. Trajectory analysis was performed using GROMACS built-in tools to evaluate conformational changes, root mean square deviation (RMSD), radius of gyration (Rg), root mean square fluctuation (RMSF), and the number of hydrogen bonds formed between the ligand and receptor.

### Measurement of endogenous IAA levels

Wild-type *Arabidopsis* seedlings treated with varying concentrations of ZR1 (0, 5, 7.5, and 10 μM) for 7 days were harvested, flash-frozen in liquid nitrogen, and ground into a fine powder. Approximately, 0.15 g of the powder was homogenized in 1.35 ml of ice-cold phosphate-buffered saline (PBS, 0.01 M, pH 7.4) on ice. The homogenate was centrifuged at 5000 × g for 15 min at 4°C, and the supernatant was collected for analysis. IAA content was quantified using a commercial ELISA kit (Jiliide, Wuhan, China, Cat. No.: J38986) according to the manufacturer's instructions. Five independent biological replicates were performed for each treatment.

### Antioxidant enzyme activity, GSH content, GSSG content assays

To assess antioxidant enzyme activity and GSH/GSSG content, root samples treated with varying concentrations of ZR1 for 7 days were ground in liquid nitrogen. Exactly 0.1 g of the frozen powder was homogenized with 1 ml of ice-cold extraction buffer provided with the assay kits. The activities of superoxide dismutase (SOD), catalase (CAT), and peroxidase (POD), as well as the contents of GSH and GSSG, were then determined using commercial assay kits (Solarbio, Beijing, China; Cat. Nos. BC5165, BC0095, BC1175, and BC1185) according to the manufacturers' protocols. Three independent biological replicates were performed for each treatment.

### DAB and NBT staining

*Arabidopsis* Col-0 seedlings with 1-cm-long roots were transferred to 1/2 MS medium supplemented with varying concentrations of ZR1 (0, 5, 7.5, and 10 μM) and cultivated for 7 days. For histochemical detection of ROS, the roots were subsequently stained. Superoxide anions were detected by immersing roots in a 2-mM nitroblue tetrazolium (NBT) solution prepared in 20 mM phosphate buffer (pH 6.1) for 20 minutes, following the previous method [58]. H_2_O_2_ accumulation was visualized by incubation in a 0.1-mg/ml DAB solution in 50 mM Tris–HCl buffer (pH 5.0) for 6 hours, as described by Zimmermann *et al.* [59]. After staining, DAB-stained roots were rinsed with 70% ethanol for 10 min to remove excess dye, whereas NBT-stained roots were observed directly. Staining intensities, corresponding to ROS levels, were assessed using DIC microscopy and quantified with ImageJ software. Each experiment was performed with 10 seedlings per treatment group. Due to inherent variability in reagent batches and staining duration, the DAB and NBT staining methods were used exclusively for comparative quantification of ROS levels in this study.

### Statistical analysis

Statistical analyses were conducted using two-tailed Student's t-tests to assess significant differences between experimental groups. All experiments were performed with a minimum of three independent biological replicates, which demonstrated reproducible results. Data are presented as mean ± standard error of the mean (SEM). Statistical significance was indicated as follows: ^*^P < 0.05, ^**^P < 0.01, and ^***^P < 0.001.

## Supplementary Material

Web_Material_uhag185

## Data Availability

The raw sequencing data have been deposited in the Genome Sequence Archive of the National Genomics Data Center under accession number CRA012700 and are publicly accessible at https://ngdc.cncb.ac.cn/gsa. All data are available within this article and its supporting information.
