# Supplementary material for: Panax-derived Zingibroside R1 exerts allelopathic effects on root architecture by modulating the auxin transport pathway and oxidative stress responses
Source: Hortic Res. 2026 May 8;13(10):uhag185. doi: 10.1093/hr/uhag185 (PMC13626713; doi:10.1093/hr/uhag185)
Supplement: Supplementary file 1 [file web_material_uhag185.zip › Supplementary figures.docx]

**Figure S1.** Systematic evaluation of the allelopathic potential of twelve *Panax*-derived triterpenoid saponins on ginseng root architecture and development, including Mock (DMSO) (A), 20(R)-Notoginsenoside R2 (B), Notoginsenoside R1 (C), Araloside A (D), 20(S)-Notoginsenoside R2 (E), Chikusetsusaponin IVa (F), Zingibroside R1 (G), Ginsenoside Rb2 (H), Ginsenoside Rb3 (I), Ginsenoside Rf (J), Ginsenoside Ro (K), Ginsenoside E (L), and Ginsenoside Rd (M). Scale bar = 1 cm.

**Figure S2.** Systematic evaluation of the allelopathic potential of twelve *Panax*-derived triterpenoid saponins on Arabidopsis root architecture and development, including Mock (DMSO) (A), 20(R)-Notoginsenoside R2 (B), Notoginsenoside R1 (C), Araloside A (D), 20(S)-Notoginsenoside R2 (E), Chikusetsusaponin IVa (F), Zingibroside R1 (G), Ginsenoside Rb2 (H), Ginsenoside Rb3 (I), Ginsenoside Rf (J), Ginsenoside Ro (K), Ginsenoside E (L), and Ginsenoside Rd (M). Scale bar = 1 cm.

**Figure S3.** Identification of specific triterpenoids from *Panax* with allelopathic activity on *A. thaliana* root growth. (A) Representative images showing root phenotypes of wild-type *A. thaliana* (Col-0) following 7-day exposure to 0 μM (Mock), 10 μM, and 50 μM of Chikusetsusaponin IVa. Scale bar = 1 cm. (B) Temporal progression of root elongation in Col-0 seedlings under 0 μM (Mock), 10 μM, and 50 μM of Chikusetsusaponin IVa over a 7-day period. (C) Root apex architecture in Col-0 seedlings visualized by DIC microscopy following 7-day treatment with 0 μM (Mock), 10 μM, and 50 μM of Chikusetsusaponin IVa. Scale bar = 50 μm. (D) Representative images showing root phenotypes of wild-type Col-0 seedlings following 7-day exposure to 0 μM (Mock), 10 μM, and 50 μM of Zingibroside R1. Scale bar = 1 cm. (E) Temporal progression of root elongation in Col-0 seedlings under 0 μM (Mock), 10 μM, and 50 μM of Zingibroside R1 over a 7-day period. (F) Root apex architecture in Col-0 seedlings visualized by DIC microscopy following 7-day treatment with 0 μM (Mock), 10 μM, and 50 μM of Zingibroside R1. Scale bar = 50 μm. (G) Representative images showing root phenotypes of wild-type Col-0 seedlings following 7-day exposure to 0 μM (Mock), 10 μM, and 50 μM of Calenduloside E. Scale bar = 1 cm. (H) Temporal progression of root elongation in Col-0 seedlings under 0 μM (Mock), 10 μM, and 50 μM of Calenduloside E over a 7-day period. (I) Root apex architecture in Col-0 seedlings visualized by DIC microscopy following 7-day treatment with 0 μM (Mock), 10 μM, and 50 μM of Calenduloside E. Scale bar = 50 μm.

**Figure S4.** Effect of ZR1 treatment on endogenous IAA levels. n = 5.

**Figure S5.** The phenotypic effects of ZR1 on the auxin signaling mutants *aux1* and *pin2*.

**Figure S6.** Molecular docking of ZR1 with the IAA signaling protein TIR1. (A) Docking mode of ZR1 into the active site of TIR1. (B) Interactions between ZR1 and amino acid residues in the active site of TIR1 (3D view). (C) Interactions between ZR1 and amino acid residues in the active site of TIR1 (2D view).

**Figure S7.** Molecular docking of ZR1 with the IAA signaling protein IAA7. (A) Docking mode of ZR1 into the active site of IAA7. (B) Interactions between ZR1 and amino acid residues in the active site of IAA7 (3D view). (C) Interactions between ZR1 and amino acid residues in the active site of IAA7 (2D view).

**Figure S8.** Molecular docking of ZR1 with the IAA signaling protein ARF7. (A) Docking mode of ZR1 into the active site of ARF7. (B) Interactions between ZR1 and amino acid residues in the active site of ARF7 (3D view). (C) Interactions between ZR1 and amino acid residues in the active site of ARF7 (2D view).

**Figure S9.** Molecular docking of ZR1 with the IAA signaling protein ARF19. (A) Docking mode of ZR1 into the active site of ARF19. (B) Interactions between ZR1 and amino acid residues in the active site of ARF19 (3D view). (C) Interactions between ZR1 and amino acid residues in the active site of ARF19 (2D view).

**Figure S10.** Molecular dynamics simulation results of the ZR1–ARF7 ligand–receptor complex. (A) The Root Mean Square Deviation (RMSD) of the ZR1–ARF7 complex. (B) Radius of Gyration (Rg) of the ZR1–ARF7 complex. (C) Root Mean Square Fluctuation (RMSF) of the ZR1–ARF7 complex. (D) The number of hydrogen bonds of the ZR1–ARF7 complex.

**Figure S11.** Molecular dynamics simulation results of the ZR1–IAA7 ligand–receptor complex. (A) The Root Mean Square Deviation (RMSD) of the ZR1–IAA7 complex. (B) Radius of Gyration (Rg) of the ZR1–IAA7 complex. (C) Root Mean Square Fluctuation (RMSF) of the ZR1–IAA7 complex. (D) The number of hydrogen bonds of the ZR1–IAA7 complex.

**Figure S12.** Molecular dynamics simulation results of the ZR1–ARF19 ligand–receptor complex. (A) The Root Mean Square Deviation (RMSD) of the ZR1–ARF19 complex. (B) Radius of Gyration (Rg) of the ZR1–ARF19 complex. (C) Root Mean Square Fluctuation (RMSF) of the ZR1–ARF19 complex. (D) The number of hydrogen bonds of the ZR1–ARF19 complex.

**Figure S13.** Molecular docking of IAA with TIR1 protein. (A) Docking mode of IAA into the active site of TIR1. (B) Interactions between IAA and amino acid residues in the active site of TIR1 (3D view). (C) Interactions between IAA and amino acid residues in the active site of TIR1 (2D view).

**Figure S14.** Molecular docking of IAA with ARF7 protein. (A) Docking mode of IAA into the active site of ARF7. (B) Interactions between IAA and amino acid residues in the active site of ARF7 (3D view). (C) Interactions between IAA and amino acid residues in the active site of ARF7 (2D view).

**Figure S15.** The expression levels of *TIR1* and *ARF7* in roots of ZR1‑treated Arabidopsis.

**Figure S16.** Zingibroside R1 impact on Arabidopsis callus induction. (A) Arabidopsis root callus induction with different concentration of ZR1 after the seedlings grew to 1.0 cm. n = 10. (B) Morphological characterization of primary root callus under varying concentrations of ZR1 treatment. n = 20. (C) Statistical evaluation of dose-dependent changes in root callus formation area following ZR1 exposure. n = 20.

**Figure S17**. Volcano plot visualization of differentially expressed genes (DEGs) in *A. thaliana* roots following ZR1 treatment. (A) The volcano map of DEGs between 5 µM ZR1 and Mock. (B) The volcano map of DEGs between 10 µM ZR1 and Mock. (C) The volcano map of DEGs between 5 µM and 10 µM ZR1. The abscissa represents the change in gene expression fold.

**Figure S18**. Comparative Gene Ontology (GO) enrichment analysis among pairwise comparisons of 5 µM ZR1 vs 0 µM ZR1, 10 µM ZR1 vs 0 µM ZR1, and 10 µM ZR1 vs 5 µM ZR1.

**Figure S19**. Schematic diagram of the different regions of the root tip, including the meristematic zone, elongation zone, and maturation zone.
