# Supplementary material for: Panax-derived Zingibroside R1 exerts allelopathic effects on root architecture by modulating the auxin transport pathway and oxidative stress responses
Source: Hortic Res. 2026 May 8;13(10):uhag185. doi: 10.1093/hr/uhag185 (PMC13626713; doi:10.1093/hr/uhag185)
Supplement: Supplementary file 1 [file web_material_uhag185.zip › Supplementary table.docx]

**Table S1.** Statistical analysis of lateral root density following combined treatment with ZR1 and NPA. n=10.

| **Group** | **Lateral root number** | **Lateral root density** |
| --- | --- | --- |
| ZR1 0 μM | 12.9±2.4 | 2.1±0.4 |
| ZR1 5 μM | 9.4±1.8 | 1.8±0.5 |
| ZR1 7.5 μM | 6.9±1.6 | 2.1±0.4 |
| ZR1 10 μM | 7.9±2.5 | 3.6±1.2 |
| NPA 10 μM | 0±0 | 0±0 |
| NPA 50 μM | 0±0 | 0±0 |
| ZR1 5 μM+NPA 10 μM | 0±0 | 0±0 |
| ZR1 5 μM+NPA 50 μM | 0±0 | 0±0 |
| ZR1 7.5 μM+NPA 10 μM | 0±0 | 0±0 |
| ZR1 7.5 μM+NPA 50 μM | 0±0 | 0±0 |
| ZR1 10 μM+NPA 10 μM | 0±0 | 0±0 |
| ZR1 10 μM+NPA 50 μM | 0±0 | 0±0 |
